# Implementing regular physical activity for older individuals in the family strategy program using the RE-AIM framework to ensure feasibility and sustainability: EISI study protocol

**DOI:** 10.1016/j.conctc.2024.101311

**Published:** 2024-05-24

**Authors:** Saulo Vasconcelos Rocha, Clarice Alves dos Santos, Ariani França Conceição, Bruna Maria Palotino-Ferreira, Danilo Barbosa Morais, Félix Salvador Chavane, Carolina Rego Chaves Dias, André Luís Lacerda Bachi, Rui Mendes, Sónia Brito-Costa, Sofia Silva, Guilherme Eustáquio Furtado

**Affiliations:** a State University of Southwest Bahia, Health Sector II, Av. José Moreira Sobrinho, S/n -Jequiezinho, 45205-490, Jequié, Bahia, Brazil; b State University of Feira de Santana, Department of Health, Av. Transnordestino, S/n- Novo Horizonte, 44036-900, Feira de Santana, Bahia, Brazil; c Eduardo Mondlane University, Higher School of Sport Sciences, Av. Julius Nyerere, N. 3453- Main Campus, Maputo, Mozambique; d Post-Graduation Program in Health Sciences, Santo Amaro University, Rua Prof. Enéas de Siqueira Neto, 340, 04829-300, São Paulo, Brazil; e Polytechnic Institute of Coimbra, Coimbra Education School, Rua Dom João III - Solum, 3030-329, Coimbra, Portugal; f SPRINT - Sport Physical Activity and Health Research INvation cenTer, Rua Dom Joao III – Solum, 3030-329, Coimbra, Portugal; g Applied Research Uni in Sport Sciences, Coimbra Education School, Rua Dom Joao III – Solum, 3030-329, Coimbra, Portugal; h Polytechnic Institute of Coimbra, Applied Research Institute, Rua da Misericórdia, Lagar Dos Cortiços – S. Martinho Do Bispo, 3045-093, Coimbra, Portugal; i NED - Center for Research and Innovation in Education, Polytechnic Institute of Coimbra Education School, Rua Dom Joao III – Solum, 3030-329, Coimbra, Portugal; j Research Centre for Natural Resources Environment and Society (CERNAS), Polytechnic Institute of Coimbra, Bencanta, 3045-601, Coimbra, Portugal

**Keywords:** Sedentary behavior, Public health, COVID-19, Vaccination, Active lifestyle, Sustainable development goals

## Abstract

The EISI study protocol aims to address the low participation rate in physical exercise programs among older individuals, emphasizing its significance as a non-pharmacological therapeutic approach for overall health and increased physical activity. The objectives include implementing physical activity (PA) and educational health programs in Jequié, Bahia, Brazil, targeting the Family Health Strategy population to enhance local physical activity levels among older individuals. The study also seeks to evaluate the program's feasibility, safety, and sustainability for large-scale implementation, along with assessing its impact on immune and inflammatory response biomarkers to the SARS-CoV virus, as well as physical-functional and brain health. Participants, aged 60 or above, will be divided into two groups: multicomponent exercise (MCE) and behavioral change interventions (BCI). The study employs a mixed-method approach, utilizing a non-randomized controlled short-term pathway model for a 4–8 weeks of pilot study and 16-week intervention impact assessment. Data collection encompasses various aspects such as sociodemographic information, mental health, physical fitness, fall risk, functional capacity, anthropometric measurements, hemodynamic assessment, habitual physical activity, and health-related quality of life. Blood and saliva samples are collected for cytokine and antibody biomarker analysis related to SARS-CoV immunity. Pre- and post-intervention evaluations for both groups will be conducted, with the hypothesis that MCE will yield more favorable responses compared to BCI. The study's holistic approach, including the assessment of feasibility, safety, and sustainability, aims to contribute to achieving Sustainable Development Goals (SDG) 3 and SDG 9 b y promoting accessible and sustainable healthcare initiatives for older individuals. This research aligns with global efforts to enhance health and well-being, emphasizing the importance of regular exercise in the aging population.

## 1 Introduction

The global aging of the population has been increasing considerably and progressively, which can be attributed to a rise in life expectancy, declining birth rates, improved control of infectious diseases (through immunization), and chronic-degenerative conditions [1]. In the Brazilian population, aging follows the global trend and bears implications for public health [2].

A higher incidence and prevalence of non-communicable chronic diseases (NCDs) have been recorded among the older population, including cardiovascular diseases, diabetes, respiratory diseases, and cancer [3]. These NCDs are the leading cause of morbidity and mortality in the country, and they are largely associated with lifestyle habits such as smoking, physical inactivity (PA), and high-calorie diets [4]. This concerning global scenario demands the integration of public policies for the prevention and control of NCDs, which are based on the adoption of an active lifestyle [5].

Aging is a risk factor NCDs [6], and the literature consistently describes the effects of disease accumulation on health, including musculoskeletal dysfunction, resulting in a reduction in functional capacity [7]. This is accompanied by reductions in physical fitness, such as grip strength, endurance, and muscular strength [8] and a decrease in the ability to sustain exercise or engage in moderate to vigorous PA in advanced ages in a sustainable manner [9]. In turn, compromises in physical health are associated with an increase in sedentary behavior, reduced levels of PA (LPA), and a higher incidence of NCDs, creating a feedback loop [10]. On the other hand, recent evidence has revealed that physically active older adults exhibit higher levels of physical performance, lower risk of NCD accumulation, early hospitalization, and mortality [11].

The decrease in LPA has been associated with a decline in metabolic and immune health in older adults, which can have a negative impact on mental health [12]. Recent studies have consistently reported that low LPA can lead to increased adiposity, insulin resistance, and chronic inflammation [13], all of which are directly linked to the development of metabolic diseases and contribute to oxidative stress in the body [14], which can negatively affect the immune system [15,16]. On the other hand, investigations have demonstrated that regular physical exercise is associated with improved metabolic and immune health and it is considered a “gold standard” treatment for the prevention, mitigation, and management of mental health conditions such as depression and anxiety [17].

In Brazil, assessments of the national public health surveillance system (VIGITEL) between 2011 and 2016 found no significant increases in leisure-time PA, and reported that only 22 % of adults aged 65 years or older met the recommended LPA [18,19]. It is evident that LPA decrease with age, confirming that older Brazilians are a vulnerable group to physical inactivity and NCDs [20,21]. Adding to this, the recent COVID-19 pandemic has imposed significant social changes that have affected people's lifestyles and behaviors, especially among the older population [22]. During lockdown measures, older individuals with NCDs increased their sedentary time and decreased the minutes of walking or other moderate PA per week [23].

Structured exercise programs for groups with a community-based approach have proven to be an excellent way to increase activity levels and physical fitness among the older population [24]. The literature suggests that supervised and structured exercise programs provide more efficient health benefits for older adults compared to unsupervised [25]. However, studies have shown a progressive decrease in adherence to exercise programs over time [26]. This decline in adherence is associated with multiple barriers identified in previous literature, including age, location (or region), gender, socioeconomic conditions, and individual perception (i.e., pleasure, satisfaction, enjoyment), which vary among the population [27].

Recently, there has been discussion about the most effective strategies to increase adherence and reduce barriers to regular exercise in programs targeting geriatric populations, aiming to ensure the sustainability of PA programs [28]. Achieving sustainability in PA programs is a crucial challenge for promoting health in geriatric populations [29]. Several theoretical models are currently utilized to assess the effectiveness and sustainability of public health interventions, including PA programs [30,31]. One example is the RE-AIM model, which encompasses five dimensions: reach, efficacy, adoption, implementation, and maintenance [32]. Recent studies have demonstrated the efficacy of the RE-AIM model in assessing and improving the sustainability of PA programs for geriatric populations, and it has even been used as a tool to develop interventions in the field of PA that can be successfully implemented in real-world settings [33].

The adoption of the RE-AIM model can assist in identifying the strengths and weaknesses of physical activity programs and inform strategies to enhance their sustainability [32]. Recognizing the need for the implementation of public policies targeting populations with low LPA, it is essential to consider intersections such as gender, economic status, and racial identity, acknowledging the social inequality surrounding physical exercise practices in Brazil [34]. Furthermore, the adoption of the RE-AIM model to evaluate the sustainability of PA programs for older adults is of paramount importance in achieving the Sustainable Development Goals (SDGs) set forth by the United Nations [35]. By identifying strengths and comprehending the programs, it becomes possible to develop strategies to improve their sustainability, ensuring that the promotion of physical activity is effective and enduring [36].

The Intervention Study Protocol in Older Health (EISI-SP) aims to implement a physical exercise program for users of the Family Health Strategy in the municipality of Jequié, Bahia, Brazil, with the purpose of increasing LPA in the local population. As a secondary objective, this program will be assessed using the RE-AIM model to determine its feasibility and sustainability for large-scale implementation. Furthermore, at the end of the program, which will span 8 weeks of intervention with a multicomponent exercise and behavioral change approaches, the impact on several metabolic, immune, functional physical fitness, psychological well-being, and mental health indicators will be evaluated.

## 2 Methods

### 2.1 Study design

The EISI-SP will be a community intervention study, using a non-randomized controlled (nRCT), and short-term pathway model lasting 8–10 weeks [37], with two arms comprised physical exercise group versus control group with lifestyle behavioral change intervention. A pre- and post-design will be employed to assess the impact of the EISI program on several physical, metabolic, cognitive, and immunological function variables in the quantitative analysis. Additionally, a mix method of qualitative-quantitative approach will be conducted to evaluate the components of the RE-AIM model. This study protocol was developed based on the SPIRIT guidelines too [38].

### Ethical statement

The EISI-SP has been approved by the Ethical Committee of the State University of Southwest Bahia with CAEE code 60974222.2.0000.0055. This study protocol adhered to the Brazilian Resolution (466/2012) of the National Council of Health on ethics in research with human participants [39], followed the guidelines for ethics in scientific experiments in exercise science research [40], and complied with the guidelines for research with human subjects of the Helsinki Declaration [41].

### 2.2 - Eligibility criteria for participants

**Fig. 1:**
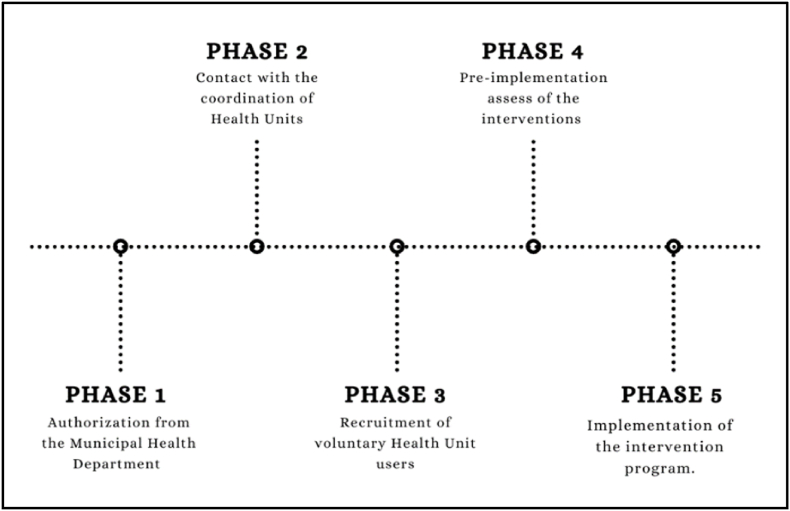
**–** Timeline of the EISI study implementation.

Fig. 1 indicating the steps of the study implementation. Individuals registered in the Family Health Strategy (FHS) of the urban area of Jequié-Bahia, Brazil, were invited to participate in the study. Potential participants met the following inclusion criteria: a) not participating in structured physical exercise programs; b) physically independent (able to move independently); c) aged 60 years or older; d) of both genders; e) no diagnosis of SARS-CoV-2 virus within the last 3 months; f) absence of cognitive impairment recorded in medical records; g) completed COVID-19 vaccination strategy. The following exclusion criteria were applied: a) uncontrolled hypertension; b) acute or severe heart failure; c) acute or severe respiratory failure; d) uncontrolled postural hypotension; e) decompensated or uncontrolled diabetes mellitus; f) recent fractures; g) cognitive impairments that could affect test performance; h) any other condition that hinders the completion of physical activity. All clinical health information will be obtained through consultation with the available health records in the ESF.

A minimum sample size of 20 participants per group will be recruited, which is sufficient to detect significant changes considering a statistical effect size of p = 0.05, as established take into account previous similar study [42]. However, considering a predicted adherence rate of 70 % to the program, a minimum sample size of n = 24 for both the intervention and control groups is justified.

### 2.3 Precautions

The exercise sessions' instructor did not participate in data collection procedures. Trained research personnel conducted the collection of saliva, blood, and global health assessments. The principal investigators organized the assessment of psychometric and cognitive scales, as well as the physical-functional fitness battery, which was administered by specialists and co-investigators of the research team. To minimize procedural differences, the same evaluators performed data collection, completed both baseline and follow-up questionnaires, and conducted the physical-functional fitness battery tests. To accommodate participants who face difficulties in moving outside of their neighborhood for study participation, the classes will be conducted in suitable spaces within the Family Health Units (USFs).

### 2.4 Assessments

This evaluation phase involves initiating contact with the medical staff of the Family Health Strategy (FHS) to acquire pertinent information regarding participants' medical history and current health, factors that may impact their engagement in the exercise programs. To conduct the initial assessments, a standardized data collection instrument was employed. Participants completed this instrument two weeks prior to the program's commencement and one week following its conclusion. The comprehensive data collection instrument comprised eight sections, encompassing: a) socioeconomic and demographic information; b) lifestyle habits; c) physical-functional status; d) health status questionnaire; e) mental well-being and quality of life; f) questionnaire on Covid-19 infections; g) anthropometric measurements; h) fall risk assessment; and i) feasibility and sustainability of interventions. Application of these questionnaires during this phase was carried out by a team consisting of undergraduate and postgraduate students, physical education professionals, and university professors.

#### 2.4.1 Socioeconomic and demographic information

The following demographic and personal information based on will be collected [43]: gender (male/female), age (continuous variable in complete years), marital status (married, cohabiting, single, divorced/separated, widowed), education level (never attended school, reads and writes their name, primary school: 1st to 4th grade, lower secondary school: 5th to 8th grade, high school: 1st to 3rd year, higher education: completed, incomplete, 3rd to 4th year), race/ethnicity (white, yellow (oriental), mixed race, indigenous origin, black, unknown), number of living children (variable in Arabic numerals), housing characteristics (living with others, living alone, no response, unknown), religion (Catholic, Protestant, Jewish, Spiritus/Kardecist, Umbanda, Other), average per capita family income (quantified in Brazilian Reais R$, *2022: minimum wage R$ 1302.00), and occupation (yes or no).

#### 2.4.2 Brain health

For the assessment of cognitive status, the Brazilian version Mini-Mental State Examination (MMSE) will be employed. The MMSE evaluates five cognitive domains on a 30-points (pts) scale, including orientation, immediate recall, attention and calculation, delayed recall, and language [44]. Participants will be classified into cognitive profiles using the criteria described by Mungas: a) Severe Cognitive Impairment [01 to 09 pt]; b) Moderate Cognitive Impairment [10 to 18 pt]; c) Mild Cognitive Impairment [19 to 24 pt]; d) Normal Cognitive Profile [ 25 to 30 pt]. The World Health Organization's Self Report Questionnaire (SRQ-20) will be utilized for screening common mental disorders (CMDs) [45]. Validation studies in Brazil have demonstrated the SRQ-20's effectiveness in identifying potential CMDs, and the cutoff point of seven and five positive responses will be adopted to classify individuals, as having or not having CMDs [46].

#### 2.4.3 Physical fitness

The Short Physical Performance Battery (SPPB) will be used to evaluate physical function through three tests [47]: 1) Balance Test: Participants will be assessed in three positions - feet side by side, semi-tandem, and tandem - with the evaluator measuring the time they can maintain each position. Scores range from 0 to 2.2) 4-Meter Gait Speed Test: Participants will walk the distance three times, and the fastest time will be recorded. Scores range from 0 to 4.3) Sit-to-Stand Test: Participants will perform sit-to-stand movements for 30 s, with scores ranging from 0 to 4 based on the number of repetitions completed. The total SPPB score will be the sum of the scores from all three tests, ranging from 0 to 12. Participants will be classified as having poor (0–3), low (4–6), moderate (7–9), or good (10–12) physical capacity based on their total score [48].

#### 2.4.4 Risk of falls

The risk of falls will be evaluated using three tests: i) a one question where participants will report if they had recent falls [49]; ii) the Timed Up and Go (TUG) test, measuring the time taken to rise from a chair, walk [50]; iii) and sit back down; and the 6-Meter Gait Speed Test, assessing walking speed. These tests will help identify individuals with a moderate risk of falls based on specific time cutoffs. Additionally, participants will have the option to choose between the 6-min walk test or the 2-min stationary march to evaluate aerobic endurance. The assessments require simple instruments such as a chair, measuring tape, and stopwatch, making them feasible for the study's participants [51].

#### 2.4.5 Daily life functional tasks

Functional capacity related to activities of daily living will be assessed using the Functional Activities Questionnaire [52]. This instrument evaluates instrumental activities of daily living such as managing personal finances, cooking, and understanding one's environment, among others. The questionnaire consists of 10 questions, and each question allows the interviewee to score from 0 to 3 points. The adopted cutoff points are: 0 to 4 points = independent and ≥5 points = dependent. The questionnaire will be answered by the elderly participants, provided they do not exhibit cognitive impairments [53].

#### 2.4.6 Daily life functional tasks

Anthropometric measures will follow standard procedures in accordance with guidelines cited in previous studies [54]. The measurements will be conducted in a separate room to provide privacy to the participants. Body mass weight will be determined using a portable digital scale (Omron®) with a precision of 0.1 kg. Waist, hip, and calf circumference will be measured using a retractable tape measure (Sanny®) with a precision of 0.1 cm. Stature will be determined using a portable stadiometer (Sanny®) with a precision of 0.1 cm.

#### 2.4.7 General health status

Information on self-reported diseases related to the circulatory, respiratory, and musculoskeletal systems, medication use, and lifestyle habits such as alcohol consumption, tobacco use, and dietary patterns will be collected. In addition, will be measured using the digital sphygmomanometer by Omron® (HEM 705CPINT) before and after the intervention [55]. Participants will be asked to rest for 5 min in a seated position, and all measurements will be taken on the right arm of the participants [56,57].

#### 2.4.8 Levels of physical activity

To assess mean daily physical activity (MDPA) in the elderly population, we will use the adapted and validated International Physical Activity Questionnaire (IPAQ) [58]. The questionnaire includes 15 questions in five domains, covering various dimensions of physical tasks, including work-related, transportation, household chores, recreational activities, and sitting time [59]. The total weekly physical activity will be calculated according to WHO guidelines, with individuals classified as active if they engage in 150–300 min of moderate and/or vigorous activity per week [60]. This comprehensive assessment aims to provide valuable insights into the levels of physical activity among the elderly participants and contribute to understanding their overall activity patterns and sedentary behavior for health promotion purposes [61].

#### 2.4.9 Health-related quality of life

The evaluation of the Health-Related Quality of Life (HRQoL) was conducted using two general questions from the Brazilian version of WHOQOL-Bref [62], an adapted instrument composed of 26 questions based on the original WHOQOL-100 [63]. The questionnaire assesses four domains: physical, psychological, social relationships, and environment. Participants' perceptions of their health were analyzed, considering the two weeks preceding the questionnaire completion [64].

### 2.5 Biological samples

#### 2.5.1 Saliva samples

By following previous studies and standard procedures, saliva will be collected in a volume of 2 mL, using sterile 15 mL Falcon® tubes made of high-quality polypropylene to prevent analyte retention or contamination that may interfere with immunoassays [65]. Collection will always take place in the morning to minimize the circadian effect observed in some of the studied markers [66]. The collected material will then be cooled to −81 °C for further analysis. Subsequently, centrifugation at 25,000 rpm for 10 min will be performed [67]. From the obtained supernatant, 500 μL will be transferred to 1.5 mL Eppendorf microtubes and stored at −80 °C without any buffer or preservative to facilitate subsequent analyses of cytokines and specific immunoglobulins for the SARS-CoV-2 virus [68].

#### 2.5.2 Blood samples

Blood samples will be collected via venipuncture, while the participants are fasting, by a nurse, using appropriate tubes to obtain serum and/or plasma aliquots (minimum of 500 μL). The tubes will be centrifuged at 2500 rpm for 10 min at 4 °C to separate coagulated from non-coagulated blood [67]. The obtained aliquots will then be frozen at −80 °C until subsequent analysis and determination of serum markers. For the determination of cytokines (i.e. IL-6, IL-10, and TNF-alpha), concentrations will be measured in the previously stored serum and saliva at −80 °C using ELISA kits (R&D Bioscience, USA) [69], following the manufacturer's recommendations. To determine specific Immunoglobulins (IgA, IgM, and IgG) for the SARS-CoV-2 virus, concentrations will be measured in the serum and saliva samples using the “in-house” ELISA technique, adapted from the protocol described in previous studies [70].

### 2.6 Description of interventions

The interventions will be offered for 8–10 weeks, totaling 16–20 exercise sessions, with a weekly frequency of two sessions on non-consecutive days (separated by 48 h). All activities of the behavior change and health education program (G1, HEP) and the multicomponent exercise program (MEP, G2) will be delivered by two instructors. These instructors will be trained to maintain consistency in the classes based on the components of the MEP protocol and the topics discussed in the HEP program.

#### 2.6.1 – MEP intervention

**Fig. 2:**
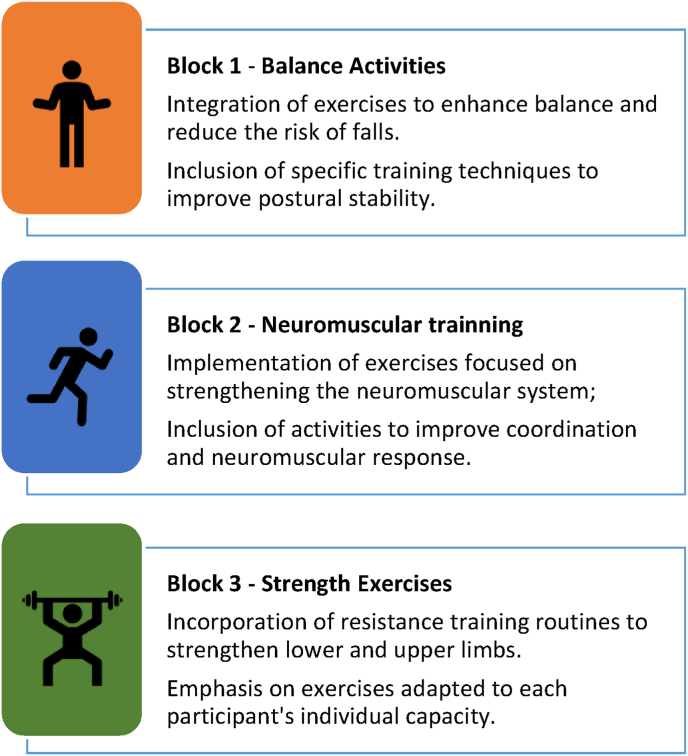
Example of exercise session of MEP applied per day, divided into time blocks.

The MEP programs will be conducted by exercise specialists. The guidelines of the ACSM for exercise prescription in older adults will be followed [71]. Additionally, the recommendations of the Vivi Frail Program will also be adhered to Ref. [72]. There will be a total of 36 sessions, with a frequency of 2 times per week, spanning a period of 4 months. Each session will last for 50 min, divided into three blocks. The first block, lasting 10 min, will consist of aerobic and balance exercises. The second block will focus on 30 min of neuromuscular exercises, and finally, the last 10 min will be dedicated to cool-down exercises with stretching routines. Examples of the exercises to be performed are described in Fig. 2.

In the first block, the exercises will be performed based on time, ranging from one to two and a half minutes of execution each. In the second block of neuromuscular exercises, three sets of exercises will be performed, stimulating basic patterns of pushing, pulling, and squatting, with repetitions ranging from 12 to 20. The rest interval will vary from 1 min to 30 s, depending on the intensity increment. To increase the load, training methods such as isometric exercises will be added at the end of each set. In the third block, stretching exercises will be performed, targeting both the upper and lower limbs, with the duration of each stretch being determined based on individual needs and goals.

#### 2.6.2 HEP intervention

Based on the “*VAMOS: Vida Ativa Melhorando a Saúde*" program, the control group of HEP received lectures on health education, physical activities, healthy eating, identifying social support for an active lifestyle, and the impacts of an active lifestyle on mental health [73]. “VAMOS” is a behavior change educational program consisting of 12 sessions (see Table 01). In the proposed intervention, a total of 4 sessions will be conducted over a period of 2 months, with 2 sessions per month every 15 days, on the same day of the week, at the same time and location HSF, lasting 60–90 min each. The 4 sessions were chosen by the intervention team for this pilot project, aiming for better adherence by having fewer lectures. The intention is that, as the project progresses, all 12 lectures on each proposed theme of the “VAMOS” content will be implemented [74].

**Table 1:** Content characterization of the HEP program based on the theoretical sessions.

1st Meeting: Active Life style	2nd Meeting: Healthy Eating	3rd Meeting: Social Support	4th Meeting: Mental Health
• How can I incorporate physical activity into my daily routine? • What perceived barriers exist for engaging in physical activity?	• What strategies can be implemented to maintain a healthy diet? • And what are the impacts of a diet high in sodium and sugar on health?	• Which individuals in my social circle can I rely on to stay more physically active? • With whom can I share my physical activity goals to enhance my network?	• What is my perception of my mental health? • How does daily physical activity positively impact mental health?

#### 2.6.3 Exercise adherence

Exercise adherence to the intervention protocol will be carefully monitored by the instructors. The levels of adherence for both the HEP and MEP groups will be calculated based on the number of attended sessions, which will be used as a co-variable in secondary analysis of the exercise outcomes. In case of an absence from two consecutive classes, the instructors and research team staff will proactively motivate and encourage the participants to maintain their attendance. It is expected that either the MEP group or the HEP control group attend sessions with a frequency of less than 70 % in both intervention programs.

#### 2.6.4 RE-AIM framework

This tool encompasses five dimensions, providing a comprehensive analysis of a health intervention's potential [32]. The Reach dimension indicates the number of potentially eligible participants for the intervention. The Efficacy/Effectiveness dimension quantifies the impact of the intervention, taking into account potential negative and positive effects. Adoption is related to the readiness of organizations and teams to initiate the program. Implementation assesses how well the proposed intervention can be applied to real-world conditions, considering planning, practices, costs, and established goals for execution. Lastly, the Maintenance dimension corresponds to the sustainability of a program over time [75].

Given this, the Reach indicators will include recruitment rates, participant characteristics, and participation rates in both the intervention. Efficacy/Effectiveness will encompass intervention group outcomes, including potential individual and collective benefits from the intervention program, as well as impacts on assessed health performances, adverse event rates, participant acceptability, and adherence. Adoption will incorporate quantitative and qualitative feedback on planning, execution, work environment(s), and team involvement. Implementation will evaluate the intended and actual duration of the intervention, proposed goals, any adaptations, as well as costs and user engagement levels. Maintenance will be assessed by both organizers and participants, considering the potential for sustained execution of the HEP and MEP programs over time.

#### 2.6.5 Sustainability

After the study execution, assessments and improvements will be made for the continuation of the intervention in the medium and long term, based on the RE-AIM model. This model provides a set of indicators capable of evaluating sustainability, directly related to SDG 3, which advocates for the promotion of health and well-being in populations. Aspects related to the internal and external validity of the intervention program will be considered, evaluated through overall intervention results and focus groups [76]. In this way, we hope for the continued application of these intervention models in other realities in Brazil and other countries, such as Portugal and Mozambique, considering the partners involved in the EISI-SP.

Furthermore, certain parameters, such as adherence based on the participants' gender, related to SDG 10 (reduction of inequalities); the importance of the exercise program for building healthier and more active cities (SDG 11); the use of sustainable practices for intervention program implementation (SDG 13), and the establishment of partnerships and collaborations with local organizations for its implementation (SDG), will be considered and verified, aiming to align with the main UN SDGs related to this type of intervention [35].

#### 2.6.6 Barriers and facilitators

In order to evaluate the facilitators and barriers to participation in the exercise program, a focus group will be conducted with the participants. This approach will allow for a more holistic and contextual understanding of the underlying issues that may impact adherence to the EISI-SP, as well as identify social, environmental, and psychological factors that can positively influence or hinder the incorporation of physical activity into their daily lives.

Following methods described in previous studies [77], sessions will be organized with small groups of participants, and a facilitator will lead the discussion, encouraging participants to express their opinions, ideas, experiences, and perceptions regarding their participation in the EISI-SP. The environment will promote interaction among participants, enabling the emergence of new ideas and collective insights on the subject. The information collected during the focus group will be qualitatively analyzed to identify patterns, trends, and relevant issues related to participation in the EISI-SP.

### 2.7 Focus group analysis

The focus group will be the qualitative approach used to evaluate the elderly and their participation in EISI-SP. The steps include planning, participant selection, script preparation, session facilitation, discussion and interaction among participants, data recording and analysis [78]. The incorporation of this methodology will provide detailed insights into the perspectives, challenges, and successes of the elderly in the program, assisting in improvements and adaptations to meet their real needs.

### 2.8 Pilot study

During a 4-week pilot study, one exercise session per week was conducted. Simultaneously, interviews, psychometric scales, and functional tests were administered to assess the subjects' conditions and evaluate the study's application methods. Throughout this period, 30-min classes (pre-training, easy-level) were provided to gain insights into the implementation of exercise programs, assess the suitability of the spaces and structures for the classes, and test the participants' rating of perceived exertion.

### 2.9 Statistical data analysis

The assumption of normality was assessed using the Kolmogorov-Smirnov test with Lilliefors' significance correction, as well as through visual inspection of normality plots. For the specific case of comparing participants in the MEP intervention programs and comparison with HEP control group individuals, who were measured twice on the same variables at baseline and after 8 weeks of intervention, we will use either an independent T-test or Wilcoxon test, depending on data assumptions. Correlations will be calculated using Spearman's rank correlations.

To determine the effect sizes, the between-subject standard deviation for each dependent variable will be used to convert changes in all variables into standardized Cohen effect sizes (ES). Following Hopkins' guidelines, effect sizes will be categorized as trivial (d ≤ 0.2), small (0.2 < d < 0.6), moderate (0.6 < d < 1.2), large (1.2 < d < 2.0), very large (2.0 < d < 4.0), and nearly perfect (d > 4.0). This will be further confirmed by correlation matrices, where all variables in a group should correlate with at least 50 %, and principal component analyses, where the first component should account for at least 70 % of the variance.

## 3 Discussion

The presented EISI-SP adopts a comprehensive approach to evaluate the effectiveness and sustainability of a PA and HBC program for the elderly in the Family Health Strategy of Jequié, Bahia, Brazil. The methodology, in addition to including a quantitative approach to assess the program's impact on multiple dimensions, also involves the use of a focus group to understand the perceptions, facilitators, and barriers faced by the elderly regarding program participation. This qualitative approach will allow for a deeper and contextualized analysis of underlying issues that may impact adherence to the program, assisting in the improvement and adaptation of interventions to meet the real needs of the community-dwelling older adults.

This study will investigate which hormonal and immunological parameters are capable of mediating the effects of exercise on mucosal immunity and inflammatory biomarkers, health-relate quality of life, psychological well-being and mental health-status. To achieve this goal, we will follow what has been produced in previous studies by our group and other similar studies, where the use of a biopsychological approach was one of the remarkable characteristics to be verified [59,69]. The ultimate goal is to develop exercise protocols that can be implemented on a large scale in the future and support disease prevention and a better quality of life.

In this regard, the implementation of EISI-SP for this population is fundamental as it will reveal the extent to which this type of intervention is viable and sustainable in the medium and long term. This study also demonstrates a strong multidisciplinary approach, as it is prudent to investigate the effects of different EISI-SP on indicators of immune health, physical-functional fitness, health-related quality of life, and psychological well-being related to mental health.

One of the objectives is also to understand if there will be a reduced dependence on health services associated with the improvement in the perception of overall health, considering that regular PA plays an important role as a non-pharmacological therapy for the treatment of geriatric syndromes [79]. During the Covid-19 pandemic, there was a reduced number of community trial programs in the in-person format [80], due to the risks and participants' uncertainty about contracting the virus. Therefore, it is expected that there will be a good adherence from the older participants, collaborating with the implementation of a structured program within the Health Units.

Previous research conducted with exercise programs has demonstrated their relevance for healthy aging and a good quality of life, reducing the incidence of chronic diseases [81]. Participation in structured exercise programs provides significant benefits to functional capacity compared to usual care, promoting a beneficial effect on cognition, muscle function, and mood state [82]. Furthermore, the implementation of exercise programs can reduce or stabilize the progression of functional limitations and mobility, as the loss of physical and cognitive functions implies a reduced ability to manage tasks independently, affecting psychological and immune health, as well as social interaction for healthy aging [83].

Considering that one of the markers of aging is chronic inflammation in the body, some studies provide information that structured physical exercises are essential for reducing chronic inflammation, improving muscle strength, balance, and fall prevention [84]. Structured exercise programs like VIVIFRAIL can improve immunological and metabolic parameters, benefiting the elderly's body and demonstrating the beneficial effect of exercise on the body's profiles, contributing to a better understanding of treatment and prevention perspectives, and advancing scientific knowledge in the field [85]. The model is based on a low-cost intervention that can be replicated in healthcare centres, being easily implementable, aiming to disseminate a new exercise prescription tool [86].

A program based on the VIVIFRAIL proposal can be considered a sustainable and effective intervention in improving and maintaining functional capacity markers in elderly individuals, combining different motor stimuli. There is also a factor with a positive impact on the person's daily life, as they report satisfaction with program results, increasing awareness of the importance of being active [33]. Moreover, this is the first study to use a proposal based on the VIVIFRAIL model to develop a physical exercise program in FHS units in Brazil, aiming to study the older individuals comprehensively, including their physical, cognitive, metabolic, and immunological aspects.

Focus groups are valuable for collecting diverse perceptions, emotions, and meanings related to specific plans, making them ideal for exploratory or evaluative research [87]. They can complement quantitative studies, enriching data analysis by capturing subjective aspects inherent in human nature. However, in interventions with MEP in the older participants, there remains a scarcity of focus group-based studies, despite the increasing use of this methodology in involving the elderly in creating and discussing content for HEP programs.

In this sense, the experience gained from their application allows for a broader understanding of the entire intervention process that one wishes to study, taking into account subjective aspects inherent in human nature that can influence outcomes and the development of future research. These aspects reveal their importance, going beyond the numerical interpretation of data, and their complementary use in quantitative research is crucial for enriching the analysis of studies.

### 3.1 Expected results

Through the implementation of EISI-SP, we hope to achieve a significant positive impact on the health and well-being of the older community-dwelling population. EISI-SP will succeed in program implementation and achieve high adherence, evaluated by the RE-AIM model. Through the qualitative approach of focus groups, the program will be adapted and improved, considering the perceptions and needs of the elderly participants. This will result in a structured physical exercise program that is feasible, sustainable, and appealing to the elderly population registered in the Family Health Strategy of Jequié, Bahia, Brazil.

Regarding the impact on global health indicators to be evaluated, it is anticipated that the exercise program will have positive and significant effects on functional capacity, health-related quality of life, mood, and psychological well-being of the elderly participants. Additionally, the program may lead to a reduction in the incidence of chronic diseases and dependence on healthcare services, contributing to healthier aging and improved quality of life.

In relation to the Sustainable Development Goals (SDGs), the EISI program is closely related to several goals, particularly SDG 3. By promoting physical activity and behavioral change, the program aims to enhance the health and well-being of the older individuals, preventing diseases and fostering a healthy and active lifestyle. Moreover, the program may also contribute to other SDGs, such as SDG 10 - Reduced Inequalities, by promoting the participation and inclusion of the in the community, and SDG 11 - Sustainable Cities and Communities, by encouraging sustainable practices in the implementation of intervention programs. Achieving these goals in real-time, the EISI study protocol can serve as a model for other regions in Brazil and other countries, contributing to the promotion of health and well-being of the elderly worldwide.

## 4 Conclusion

The EISI-SP protocol represents a comprehensive and innovative approach to promoting health and well-being among the elderly population. Combining structured exercise programs with behavioral change interventions and utilizing qualitative methods like focus groups, the study aims to create a sustainable and effective intervention that can be easily implemented in community health settings.

Through the evaluation of the intervention using the RE-AIM model, the study seeks not only to measure its success but also gain valuable insights for future improvements and broader implementation. Addressing key indicators of global health and the impact on SDG, the EISI-SP protocol has the potential to pave the way for healthier and more active aging in Brazil and beyond. Ultimately, this research contributes to the growing body of knowledge on effective exercise and behavioral interventions for elderly populations, with the potential to create a positive and lasting impact on their well-being and overall health.

## CRediT authorship contribution statement

**Saulo Vasconcelos Rocha:** Conceptualization, Methodology, Resources, Writing – original draft, Project administration, Supervision. **Clarice Alves dos Santos:** Methodology, Conceptualization, Project administration, Resources, Supervision. **Ariani França Conceição:** Methodology. **Bruna Maria Palotino-Ferreira:** Conceptualization. **Danilo Barbosa Morais:** Methodology. **Félix Salvador Chavane:** Methodology, Resources. **Carolina Rego Chaves Dias:** Methodology, Visualization. **André Luís Lacerda Bachi:** Conceptualization, Methodology, Resources, Validation. **Rui Mendes:** Methodology, Resources, Validation. **Sónia Brito-Costa:** Conceptualization, Methodology, Supervision, Writing – original draft, Writing – review & editing. **Sofia Silva:** Methodology, Resources. **Guilherme Eustáquio Furtado:** Conceptualization, Methodology, Validation, Writing – original draft, Supervision, Writing – review & editing.

## Declaration of competing interest

The authors declare that they have no competing interests.
